# Thoraco-Omphalopagus Conjoined Twins: A Case Report

**DOI:** 10.7759/cureus.50444

**Published:** 2023-12-13

**Authors:** Ishaan M Deshmukh, Pratyaksh Chhabra, Swarupa Chakole

**Affiliations:** 1 Medicine, Jawaharlal Nehru Medical College, Datta Meghe Institute of Medical Sciences, Wardha, IND; 2 Medicine and Surgery, Jawaharlal Nehru Medical College, Datta Meghe Institute of Medical Sciences, Wardha, IND; 3 Community Medicine, Jawaharlal Nehru Medical College, Datta Meghe Institute of Medical Sciences, Wardha, IND

**Keywords:** peadiatric, womb, monozygotic twin, conjoined, thoracopagus twins

## Abstract

A conjoined twin is an uncommon congenital condition that has a very high morbidity and mortality prevalence. Identical twins united in utero are known as conjoined twins. It's an uncommon occurrence that poses a special difficulty for paediatric surgeons and obstetricians. Conjoined twins are a complicated by-product of monozygotic twinning, which raises the risk of death in the womb. One of the more prevalent varieties of conjoined twins is the thoraco-omphalopagus type, in which the heart is involved in an anterior, chest-based fusion. This case involves a 26-year-old woman who was diagnosed at 19 weeks with conjoined thoraco-omphalopagus twins using ultrasonography.

## Introduction

Conjoined twins are identical monozygotic twins that do not completely separate from one another but are still partially attached due to the incomplete division of one fertilised ovum resulting in monochorionic (sharing one placenta) and monoamniotic (sharing one amniotic sac) conjoined twins [1]. It is suggested that either fission or fusion produced this state. However, the fission and fusion theories are not able to fully explain the complete spectrum of conjoined twins and do not account for every possible conjunction [2]. One percent of monochorionic twins are conjoined twins, an uncommon and complicated condition with an estimated incidence of one in 50,000 to one in 200,000 live births [3]. Despite sharing an internal organ system, conjoined twins have a poor prognosis, with a total survival rate of 25% [4]. The success of surgery to separate the hearts of thoracopagus twins is compromised by their complicated cardiac abnormalities and tendency to share hearts [5]. However, a multidisciplinary team approach, thorough preoperative assessment of the anatomy of shared organs, and rehearsals result in a favourable outcome of operable instances [6]. Since 70% of conjoined twins pass away within 24 to 48 hours of birth or suffer from a deadly congenital condition, early detection and intervention are preferable. Pregnancy termination at the earliest opportunity remains the recommended course of action [7].

## Case presentation

A 26-year-old primigravida woman with a twin pregnancy, conceived normally, presented with a comprehensive history. Some noteworthy characteristics of her medical history included no substantial pre-existing medical issues or chronic illnesses. Through standard prenatal care, the presence of two foetuses was discovered during transabdominal ultrasonography (USG), confirming her conjoined twin pregnancy. The diagnosis was made at 19 weeks gestation. The woman regularly attended her antenatal care (ANC) sessions, which enabled medical professionals to keep an eye on her health and the development of her conjoined twin pregnancy. She reported taking the prescribed amount of folic acid supplements regularly for 19 weeks, and she also reported taking calcium pills consistently during the same time. During these consultations, conversations were started regarding the special factors related to twin pregnancies, such as elevated risks and the requirement for close observation. The woman's dedication to taking nutritious supplements, such as calcium and folic acid, shows how proactive she was about protecting her developing twins' health as well as her own. Her psychological evaluation indicated a nurturing atmosphere, and she conveyed a constructive outlook toward the forthcoming challenges. 

Physical examination

A thorough assessment was conducted during the physical examination to keep an eye on the mother and the conjoined foetuses. Notable were general observations, such as general appearance and vital signs including heart rate, blood pressure, and respiration rate. The size, shape, and fundal height were measured by abdominal palpation, which also looked for any indications of soreness or pain. The particulars pertaining to conjoined twins were taken into account during the uterine examination. Doppler USG was used to track foetal heart rates in order to evaluate cardiovascular health. Palpation and USG were used to determine position and presentation. The integrity of the skin was examined for any indications of striae or straining. A cervix assessment and exclusion of premature labour were made via a pelvic examination. Regular blood pressure monitoring was done, and for more information, laboratory procedures such as complete blood count (CBC) were carried out.

USG-obstetric (anomaly scan)

Table 1 and Table 2 show the findings obtained from USG scans.

**Table 1:** Survey A: Refers to one of the twins, designated as Foetus A; B: Refers to one of the twins, designated as Foetus B bpm: beats per minute

Foetus	A	B
Presentation	Variable	Variable
Placenta	Posterior	Posterior
Liquor	Normal	Normal
Foetal activity	Foetal activity present	Foetal activity present
Common Cardiac activity	Cardiac activity present; fetal heart rate – 144bpm	Cardiac activity present; foetal heart rate – 144bpm

**Table 2:** Biometry (Hadlock) BPD: biparietal distance; HC: head circumference; FL: femur length; W: weeks; D: days

Foetus	A	B
BPD	46mm (20W)	47mm (20W 2D)
HC	166.57mm (19W 1D)	168.14mm (19W 2D)
FL	29mm (18W 5D)	30mm (19W)

Antenatal Impression (As Seen in USG)

**Figure 1:**
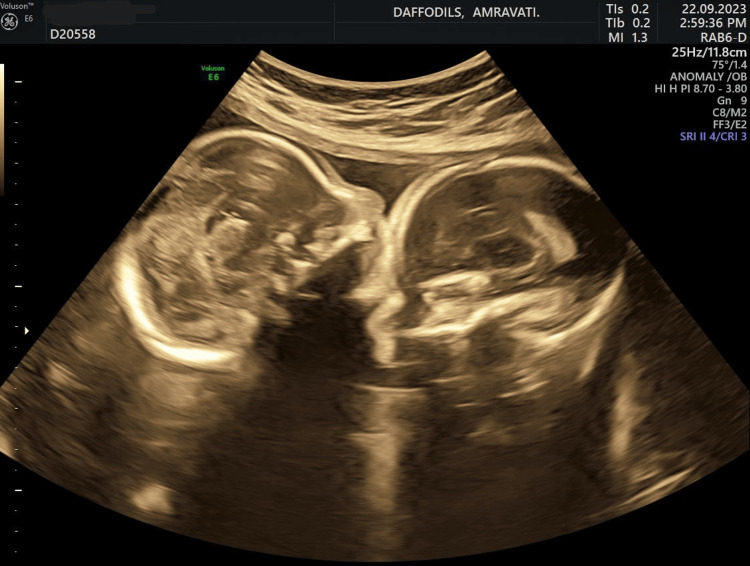
USG scan of 19-week foetuses, transabdominal route USG: ultrasonography

Several anatomical traits of the conjoined twins were discovered during the prenatal USG. Midline features in the head included a normal posterior fossa, normal lateral ventricles (AP = 5.3 mm), and a cerebellum measuring 21.4 mm. The magna cisterna was 2.9 mm in diameter and seemed typical. Foetal faces and heads were shown in different images. The neck showed no abnormalities, and the whole spine, including the spinal canal and vertebrae, was visible in both longitudinal and transverse axes. Viewed in coronal and profile views, the foetal face showed normal characteristics, with a normal interorbital distance of 11 mm, including the mouth, nose, and both orbits. Face-to-face fusion of the diaphragm, upper abdominal wall, and common sternum from the upper thorax to the umbilicus was seen between two foetuses. There were two separate hearts, and in the pericardial region, a fusion took place. There was no sign of ascites and the abdomen showed a normal situs, separate and normal stomachs, and a typical bowel pattern. The colon and rectum were observed to be distinct, despite the fact that the small intestines of both foetuses were linked. The kidneys on the right and left, together with the bladder, were all normal in the kidney, ureter and urinary bladder (KUB) region. The long bones in the extremities were normal for the gestational period. Figure 1 illustrates these features, despite the lack of finger counting.

The conjoined twins' non-varying presentation raised the possibility that they had a complicated anatomical relationship that could have influenced where they were positioned inside the uterus. The shared placenta's posterior placement and the twins' normal liquor levels implied that the amniotic fluid environment was within a healthy range and that the twins were receiving enough blood. The conjoined twins were responsive and had a working cardiovascular system as they exhibited normal cardiac activity and foetal activity, both of which had a heart rate of 144 bpm. Although the biometric measurements were within the predicted range for gestational ages, they were nonetheless significant growth indicators, and the special difficulties associated with conjoined twins made continuous monitoring of these parameters essential. The information highlights the conjoined twins' complexity and the necessity of specialised treatment and continuous observation to handle any possible issues arising from their combined anatomy. Healthcare practitioners can better comprehend the twins' developmental stage by utilising the gestational age references included in the biometric data.

Observation

**Figure 2:**
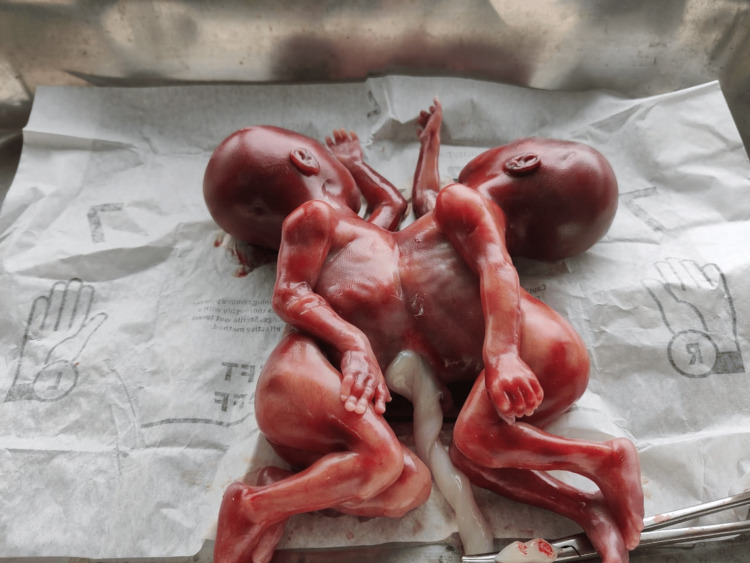
Thoraco-omphalopagus conjoined twins

Monochorionic monoamniotic twin live foetuses had a non-changing presentation of maturity at 20-21 weeks. All these features were suggestive of thoraco-omphalopagus conjoined twins. The patient was advised termination of the pregnancy as there is a very low rate of survival and those who survive are at very high risk of severe morbidity. Surgical termination was done. Figure 2 shows the thoraco-omphalopagus conjoined twins post termination.

Management

A treatment plan that took into account the intricate anatomy, possible problems, and ethical issues was developed by a multidisciplinary team of doctors that included a genetic counsellor, paediatric surgeons, and obstetricians. It was decided to end the pregnancy at 20-21 weeks following intense counselling and talks with the parents. The intended goal of the termination was to reduce any health concerns for the mother and deal with the moral dilemmas raised by the twins' similar morphology. The actual result was a meticulously thought-out and carried-out termination surgery that recognised the special issues posed by the thoraco-omphalopagus conjoined twins and ensured the primigravida received compassionate care. As part of a comprehensive approach to their treatment, the parents received continuous emotional and psychological assistance following the procedure.

## Discussion

The sites of conjunction that are most noticeable in conjoined twins' classifications are the thorax (thoracopagus), abdomen (omphalopagus), sacrum (pygopagus), pelvis (ischiopagus), skull (cephalopagus), and back (rachipagus). The most prevalent kind, according to the aspect of the embryonic disc, is thoracopagus (19%) [8]. Although the cause is unknown, it most likely results from an incomplete zygote division that happens between the thirteenth and fifteenth days following fertilisation. Conjoined twins have an overall survival rate of about 25%. With a ratio of 3:1, the disease is more common in females [9]. In contemporary obstetrics, USG can be used to diagnose prenatal conditions [10]. Any of the following classical signs identified by USG may suggest the diagnosis: inability to separate foetal bodies after careful observation, both foetal heads in the same plane, unusual cervical spine flexion backwards, no change in the relative position following manual manipulations and movements by the mother [11]. Nearly all of these results were observed in the situation, and all concerned individuals need to be aware of the symptoms connected to conjoined twins [12]. A careful USG examination is necessary to discover shared organs. Early prenatal diagnosis and evaluation may offer a window of time for the family to be counselled so that they can make an informed decision about the pregnancy and arrange for early termination if necessary, as well as for prenatal and postnatal care planning [13]. Planning should be done for prenatal monitoring and postnatal care if the parents want to carry the pregnancy to term. Separation of the twins should be done under a specialised team that includes pediatric surgeons, cardiac surgeons, anesthesiologists, intensive care specialists, neonatologists, and radiologists [14,15].

## Conclusions

The current case emphasises the morphological characteristics of thoracopagus conjoined twins that were discovered antenatally, highlighting the significance of a thorough prenatal sonographic evaluation to rule out conjoined twins in all twin pregnancies. Conjoined twins should be diagnosed as soon as possible to allow for an early abortion following marital counselling. The survival rate of conjoined twins is low, and there are currently relatively few documented successful separations in the literature. The degree to which the twins' shared organs are utilised determines the outcome of separation surgery in thoracopagus twins. The parents should be offered the option to end the pregnancy if the diagnosis is made before viability. Thoraco-omphalopagus conjoined twins is a very rare case scenario and its occurrence is very sparse. This research discussed the various intricate details that were taken care of, including problems regarding the family's emotions and other medical concerns. 
